# Histologic Assessment of Intratumoral Lymphoplasmacytic Infiltration Is Useful in Predicting Prognosis of Patients with Hepatocellular Carcinoma

**DOI:** 10.1371/journal.pone.0155744

**Published:** 2016-05-19

**Authors:** Akimasa Hayashi, Junji Shibahara, Kento Misumi, Junichi Arita, Yoshihiro Sakamoto, Kiyoshi Hasegawa, Norihiro Kokudo, Masashi Fukayama

**Affiliations:** 1 Department of Pathology, Graduate School of Medicine, The University of Tokyo, Tokyo, Japan; 2 Hepato-Biliary-Pancreatic Surgery Division, Department of Surgery, Graduate School of Medicine, The University of Tokyo, Tokyo, Japan; University of North Carolina School of Medicine, UNITED STATES

## Abstract

In the present study, we investigated the clinicopathologic significance of intratumoral lymphoplasmacytic infiltration in a large cohort of patients with solitary hepatocellular carcinoma (HCC). Based on examination of hematoxylin and eosin-stained sections, significant infiltration was defined as dense lymphoplasmacytic infiltration, either multifocal or diffuse, in 2 or more fields under low-power magnification. Of 544 cases, 216 (39.7%) were positive for significant infiltration (HCC-LI group), while 328 (60.3%) were negative (HCC-NLI group). There were no significant between-group differences in patient age, sex, or background etiology. The lower incidence of Child-Pugh stage B (*P* = 0.001) and lower level of indocyanine green retention rate at 15 minutes (*P* < 0.001) in the HCC-LI group indicated better liver function in this group. Histologically, tumors were significantly smaller in size in the HCC-LI group than in the HCC-NLI group (*P* < 0.001). In addition, prominent neutrophilic infiltration, interstitial fibrosis and tumor steatosis were significantly more frequent (*P* < 0.001) in the HCC-LI group, while tumor necrosis was significantly less frequent (*P* = 0.008). Kaplan-Meier analyses revealed that overall and recurrence-free survival were significantly better in the HCC-LI group (*P* < 0.001). Multivariate Cox regression analysis showed that intratumoral lymphoplasmacytic infiltration was independently prognostic of both overall and recurrence-free survival (*P* < 0.001), with absence of infiltration showing high Cox-hazard ratios for poor prognosis. In conclusion, intratumoral lymphoplasmacytic infiltration, as determined by assessment of hematoxylin and eosin-stained slides, was significantly associated with the clinical and pathologic features of HCC and has profound prognostic importance.

## Introduction

Hepatocellular carcinoma (HCC) is a major cancer worldwide,[1] and its incidence is expected to increase.[2] Despite recent advances in resection and ablation techniques, the recurrence rate after initial treatment remains high and the prognosis of patients with HCC is generally unfavorable.[3, 4] In addition, chemotherapy for recurrent HCC after surgical resection is relatively ineffective.[5]

To manage treatment-refractory cancers, including HCC, therapies are being designed to specifically target the immune system rather than cancer cells.[6] Immune cells have both antitumorigenic and protumorigenic activities, with the latter predominating in patients with non-eliminated, clinically detectable tumors.[7] The roles of inflammatory cell types in tumor immunity have been assessed. For example, cytotoxic T cells exert antitumor activity, whereas tumor-infiltrating macrophages and regulatory T cells repress such activity.[8] Immune-targeted therapies reinforce antitumor response or prevent inhibitory activity. The degree of immune cell infiltration has prognostic value in many types of cancer.[6] Tumor infiltrative lymphocytes (TILs) have generally been associated with favorable prognosis.[9] More specifically, different types of infiltrative lymphocytes have different prognostic values.[8]

Several studies have analyzed the prognostic significance of immune cell types in patients with HCC. For example, infiltration of CD8-positive T cells has been associated with good prognosis,[10] whereas infiltration of Foxp3-positive T cells has been associated with poor prognosis.[11, 12] However, the immunohistochemical procedures required for these analyses may be difficult to implement in routine diagnostic practice. In addition, considering that TILs have cell-type-dependent roles, it is important to determine the general roles and significance of TILs. To our knowledge, however, few studies of HCC have focused on the prognostic significance of TILs from a broad point of view. Wada et al.[13] revealed that massive lymphocyte infiltration was associated with better prognosis in patients with small HCC after surgical resection. Unitt et al.[14] showed that mild to moderate lymphocytic infiltration correlated with better prognosis after liver transplantation for HCC. These studies, however, did not provide sufficient evidence on the clinicopathologic significance of TILs in patients with HCCs treated by surgical resection, the primary curative treatment for solitary HCC of any size.[15]

In addition to lymphocyte infiltration, accompanied plasma cell infiltration has been reported in several types of cancer, including HCC [13, 16]. Though the role of infiltrative plasma cells remains controversial, a recent article described their characteristics, in particular their role in producing IgG4 [17].

In this study, we assessed intratumoral lymphoplasmacytic infiltration in more than 500 solitary HCCs by examining hematoxylin and eosin-stained (H&E) slides to precisely determine the clinicopathologic significance of intratumoral lymphoplasmacytic infiltration in HCC.

## Materials and Methods

### Patients

This retrospective analysis included consecutive patients with HCC who underwent initial surgical resection at the University of Tokyo Hospital from January 1, 1995 to December 31, 2011. Patients who underwent preoperative transarterial therapy or portal embolization were included only if a sufficient portion of the tumor remained viable. To precisely evaluate tumor prognosis, only patients with solitary HCCs, with or without intrahepatic metastasis, were included, whereas patients with multicentric HCCs[18] and those undergoing resection for liver transplantation were excluded.

### Clinical data

Evaluation of patient records was used to determine baseline demographic and clinical data, including patient age, sex, and serum data immediately before surgery; status of hepatitis virus infection; presence or absence of diabetes mellitus; and history of heavy alcohol consumption (≥ 80 g/day). All patients were regularly screened for HCC recurrence by monitoring plasma tumor markers, ultrasonography, and computed tomography. Recurrence was defined as the appearance of a new lesion with radiological features compatible with HCC. Overall survival was defined as the interval between the date of surgery and the date of death, and recurrence-free survival was defined as the interval between the date of surgery and the date of recurrence.

### Histologic review

Pathology reports and all tissue slides from all patients were reviewed. Tumor size, histologic grade, presence or absence of microvascular invasion, bile duct involvement, and intrahepatic or lymph node metastasis were re-evaluated. Other factors recorded included the presence or absence of steatosis in ≥ 5% of tumor cells, prominent interstitial fibrosis at least in one low-power field, prominent neutrophilic infiltration, and tumor necrosis. In most of these patients, these histologic factors had been evaluated in our previous study.[19]

Significant lymphoplasmacytic infiltration was defined in this study as dense multifocal or diffuse lymphoplasmacytic infiltration in two or more fields under low power magnification (×4 objective). Perinecrotic areas were excluded from the assessment of inflammatory cells. HCCs with and without significant lymphoplasmacytic infiltration were assigned, respectively, to the HCC with lymphoplasmacytic infiltration (HCC-LI) group and the HCC with no lymphoplasmacytic infiltration (HCC-NLI) group.

The fibrosis stages of background liver in patients with and without viral hepatitis were evaluated according to the METAVIR system[20] and the Nonalcoholic Steatohepatitis Clinical Research Network (NASH-CRN) scoring system,[21] respectively. Advanced fibrosis was defined as stages 3 and 4 in both the METAVIR system and NASH-CRN scoring systems. The presence or absence of steatosis (> 5%) was also recorded.

### Statistical Analysis

All statistical analyses were performed using JMP Pro 11 (SAS Institute Inc., Cary, NC, USA). Differences were considered significant at *P* < 0.05. Categorical data were analyzed using the two-sided Fisher's exact test. Quantitative variables were compared using Student’s *t* test or the Wilcoxon rank sum test, as appropriate. The Kaplan-Meier method and log-rank test were used to analyze survival. Multivariate Cox proportional hazards regression models were used to control for confounding variables. Multivariate regression analysis was performed using all clinicopathologic variables. For subanalysis (treatment-naïve vs. pretreatment patients), only clinicopathologic variables identified as significant on univariate analyses are included due to limitations regarding data size. The “liver cirrhosis” variable was excluded from multivariate analysis because all patients with cirrhosis were included in the group with advanced fibrosis.

### Ethics Statement

The study protocol was approved by the Medical Research Center Ethics Committee of the University of Tokyo. Clinical samples were collected under the University of Tokyo institutional guidelines for the study of human tissues, with all patients providing written informed consent.

## Results

### Patient overview

There were 544 patients, 418 (76.8%) males and 126 (23.2%) females, who met the inclusion criteria of the study. The mean patient age was 64.4 years (range, 13 to 85 years). Hepatitis B and C infections were noted in 116 (21.3%) and 289 (53.1%) patients, respectively, and 143 (26.3%) were negative for hepatitis virus infection. Diabetes mellitus was documented in 139 of 542 patients (25.6%), and 97 of 537 patients (18.1%) had a history of heavy alcohol consumption.

### Clinical characteristics of HCC with lymphoplasmacytic infiltration

**Fig 1:**
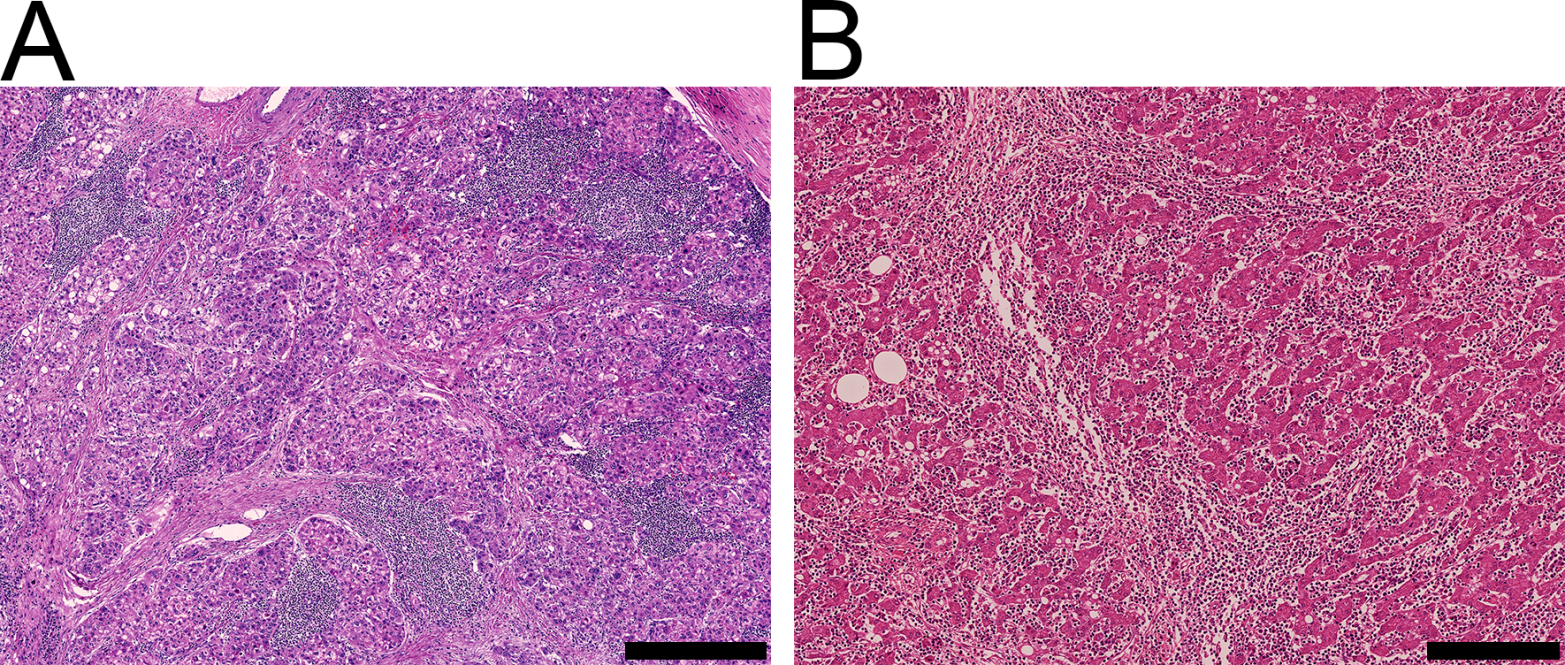
Hepatocellular carcinoma with lymphoplasmacytic infiltration. (A) Multifocal infiltration pattern. Scale bar represents 500 μm. (B) Diffuse infiltration pattern throughout the tumor, corresponding to lymphoepithelioma-like carcinoma. Scale bar represents 200 μm.

Based on our criteria, 216 (39.7%) of the 544 patients were assigned to the HCC-LI group and 328 (60.3%) to the HCC-NLI group. Lymphoplasmacytic infiltration was noted at the tumor periphery, in the interstitium, or within the deep parenchyma of the tumor (Fig 1A, S1 Digital Data. Case 1). Of 216 HCC-LIs, multifocal and diffuse patterns were observed in 162 cases (75.0%) and 54 cases (25.0%), respectively. In particular, three cases showed diffuse lymphoplasmacytic infiltration throughout the tumor, corresponding to lymphoepithelioma-like carcinoma (Fig 1B, S1 Digital Data. Case 2).

The clinical features of the HCC-LI and HCC-NLI groups are summarized in Table 1. Patient age, sex, status of hepatitis virus infection, incidence of diabetes mellitus, and history of heavy alcohol consumption did not differ significantly between the two groups. Of four serum tumor markers, the mean level of protein induced by vitamin K absence or antagonist 2 (PIVKA-2) was significantly lower in the HCC-LI group than in the HCC-NLI group (*P* = 0.022). The percent of patients with Child-Pugh stage B liver function (6.5% vs. 15.5%, *P* = 0.001) and the mean indocyanine green retention rate at 15 minutes (ICGR15) (13.3% vs.15.7%, *P* < 0.001) were significantly lower in the HCC-LI group than in the HCC-NLI group. Logistic regression analyses indicated that the absence of intratumoral lymphoplasmacytic infiltration was independently predictive of Child-Pugh Stage B and a higher ICGR15 (Table 2). Additionally, the mean serum aspartate aminotransferase (AST) concentration was significantly lower in the HCC-LI group than in the HCC-NLI group (*P* < 0.001). Significantly fewer patients in the HCC-LI group received preoperative treatment (24.5% vs. 39.3%, *P* < 0.001).

**Table 1:** Demographic and clinical features of patients with hepatocellular carcinoma with and without lymphoplasmacytic infiltration.

Clinical feature		Tumor type		
	Total N	HCC-LI	HCC-NLI	*P*
Age and Sex				
Age (years)	544			0.860
< 65	237	93	144	
> = 65	307	123	184	
Sex	544			0.535
Male	418	163	255	
Female	126	53	73	
Virus Infection				
HBs-Ag	544			1.000
Positive	116	46	70	
Negative	428	170	258	
HCV-Ab	544			0.861
Positive	288	113	175	
Negative	256	103	153	
Diabetes mellitus	542			0.616
Present	139	58	81	
Absent	403	158	245	
History of heavy alcohol consumption(≥ 80g per day)	537			0.495
Present	97	42	55	
Absent	440	174	266	
Serum tumor marker				
AFP [ng/ml, median (IQR)]	544	27 (448)	17 (234)	0.292
PIVKA-2 [mAU/ml, median (IQR)]	539	79 (680)	136 (2180)	**0.022**
CEA [ng/ml, median (IQR)]	497	3.0 (3.0)	3.4 (3.2)	0.085
CA19-9 [U/ml, median (IQR)]	490	16 (21)	17 (22)	0.631
Liver Function				
Child-Pugh stage	544			**0.001**
A	479	202	277	
B	65	14	51	
ICGR15 [%, mean ± SD]	539	13.3 ± 7.5	15.7 ± 9.7	**< 0.001**
Total protein [U/ml, mean ± SD]	544	7.1 ± 0.5	7.0 ± 0.6	0.301
Albumin [g/dl, mean ± SD]	544	3.8 ± 0.4	3.6 ± 0.4	**< 0.001**
AST [IU/l, mean ± SD]	544	43.1 ± 23.6	51.7 ± 36.5	**< 0.001**
ALT [IU/l, mean ± SD]	544	43.2 ± 28.7	48.1 ± 35.2	0.079
Total bilirubin [mg/dl, mean ± SD]	544	0.73 ± 0.32	0.77 ± 0.28	0.238
Preoperative treatment*	544			**< 0.001**
Present	182	53	129	
Absent	362	163	199	

HCC-LI, hepatocellular carcinoma with lymphoplasmacytic infiltration; HCC-NLI, hepatocellular carcinoma with no lymphoplasmacytic infiltration; AFP, alpha fetoprotein; PIVKA-2, protein induced by vitamin K absence or antagonist 2; CEA, carcinoembryonic antigen; CA19-9, carbohydrate antigen 19–9; ICGR15, indocyanine green retention rate at 15 minutes; AST, aspartate aminotransferase; ALT, alanine aminotransferase; IQR, interquartile range; SD, standard deviation.

* Including transarterial therapy and portal embolization.

**Table 2:** Risk factors for liver dysfunction (logistic multivariate analysis).

	Child Pugh stage B			ICG15 >15%		
	HR	95% CI	*P*	HR	95% CI	*P*
Tumor histology						
Lymphoplasmacytic infiltration absent (vs present)	2.438	1.248–5.010	**0.009**	1.771	1.164–2.713	**0.008**
Tumor size < 50mm (vs ≥ 50mm)	1.241	0.650–2.389	0.514	1.266	0.800–2.007	0.313
Histologic grade well and mod (vs por)	1.415	0.688–3.058	0.351	0.954	0.576–1.589	0.857
Microvascular invasion absent (vs present)	1.061	0.564–2.019	0.854	0.949	0.617–1.456	0.809
Bile duct invasion absent (vs present)	1.102	0.333–5.031	0.884	0.715	0.308–1.677	0.715
Intrahepatic metastasis absent (vs present)	1.337	0.635z2.967	0.452	0.954	0.577–1.585	0.855
Interstitial fibrosis absent (vs present)	1.012	0.553–1.872	0.968	1.178	0.780–1.779	0.436
Neutrophil infiltration absent (vs present)	1.365	0.439–6.022	0.619	1.632	0.771–3.427	0.198
Necrosis present (vs absent)	2.451	1.282–4.792	**0.007**	0.715	0.463–1.101	0.128
Steatosis present (vs absent)	1.207	0.593–2.352	0.593	1.475	0.940–2.319	0.678
Background histology						
Steatosis absent (vs present)	1.148	0.629–2.161	0.658	1.414	0.943–2.317	0.094
Advanced fibrosis present (vs absent)	2.580	1.340–5.336	**0.004**	3.602	2.379–5.548	**< 0.001**

HR, hazard ratio; CI, confidence interval; por, poorly differentiated; well, well differentiated; mod, moderately differentiated.

Advanced fibrosis corresponds to stages 3 and 4 in the METAVIR system and NASH-CRN scoring systems.

### Pathologic Features of HCC with lymphoplasmacytic infiltration

The pathologic features of HCC-LIs and HCC-NLIs are summarized in Table 3. Tumors in the HCC-LI group were significantly smaller in size than those in the HCC-NLI group (43 ± 29 mm vs. 58 ± 42 mm, *P* < 0.001), but there were no between-group differences in histologic grade, rates of microvascular invasion, bile duct invasion, or intrahepatic metastasis. Prominent neutrophilic infiltration (12.5% vs. 3.7%, *P* < 0.001), interstitial fibrosis (76.4% vs. 33.5%, *P* < 0.001) and tumor steatosis (30.6% vs. 17.7%, *P* < 0.001) were significantly more frequent in the HCC-LI group than in the HCC-NLI group, whereas tumor necrosis was significantly less frequent (49.1% vs. 60.7%, *P* = 0.008). The rates of steatosis, advanced fibrosis, and liver cirrhosis in background liver did not differ significantly between the two groups.

**Table 3:** Pathologic features of hepatocellular carcinoma with and without lymphoplasmacytic infiltration.

Pathologic feature		Tumor type		
	Total N	HCC-LI	HCC-NLI	*P*
Tumor				
Size (mm)				**< 0.001**
< 50	332	152	180	
≥ 50	212	64	148	
Histologic grade				0.152
Well differentiated	76	32	44	
Moderately differentiated	363	151	212	
Poorly differentiated	105	33	72	
Microvascular invasion				0.160
Present	286	122	164	
Absent	258	94	164	
Bile duct invasion				0.321
Present	28	14	14	
Absent	516	202	314	
Intrahepatic metastasis				0.914
Present	113	44	69	
Absent	431	172	259	
Neutrophil infiltration				**< 0.001**
Present	39	27	12	
Absent	505	189	316	
Necrosis				**0.008**
Present	305	106	199	
Absent	239	110	129	
Interstitial fibrosis				**< 0.001**
Present	275	165	110	
Absent	269	51	218	
Steatosis				**< 0.001**
Present	124	66	58	
Absent	420	150	270	
Background liver				
Steatosis (>5%)				0.452
Present	173	73	100	
Absent	371	143	228	
Advanced fibrosis*				0.165
Present	360	135	225	
Absent	184	81	103	
Liver cirrhosis				0.126
Present	208	74	134	
Absent	336	142	194	

HCC-LI, hepatocellular carcinoma with lymphoplasmacytic infiltration; HCC-NLI, hepatocellular carcinoma with no lymphoplasmacytic infiltration.

* Corresponding to stages 3 and 4 in the METAVIR system and NASH-CRN scoring systems.

### Prognosis

**Fig 2:**
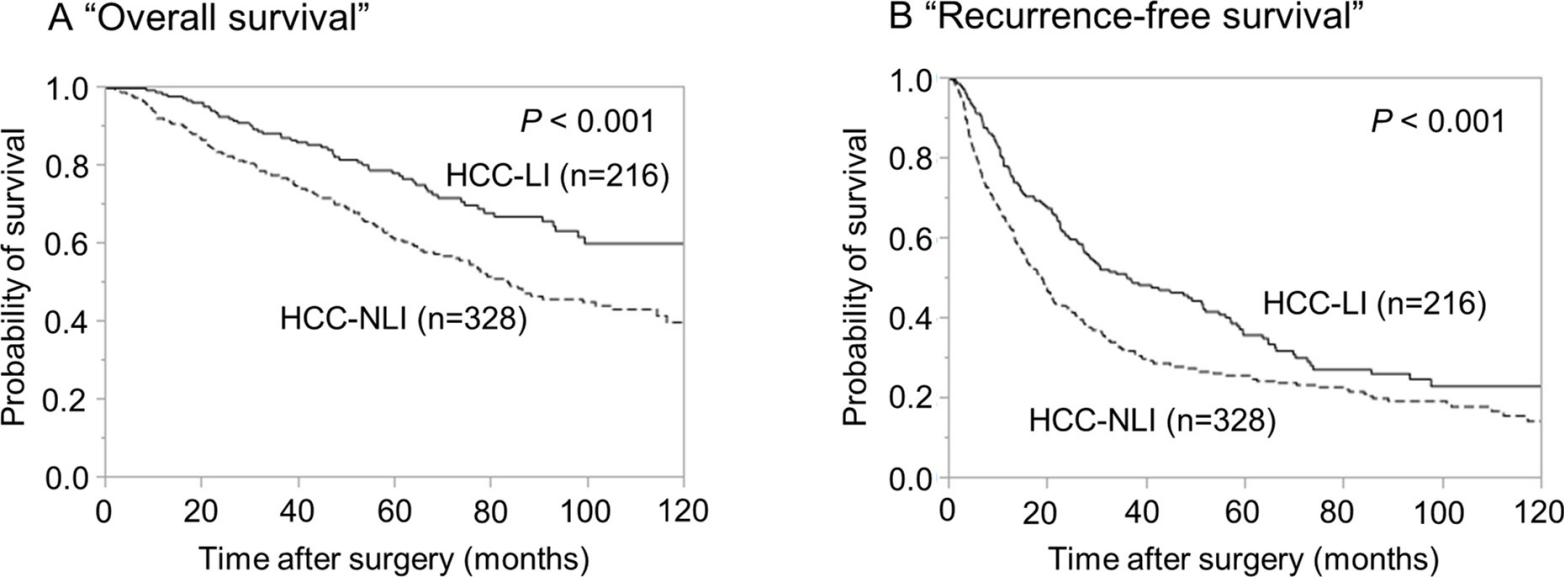
Kaplan–Meier analysis of survival in patients with HCC with and without significant lymphoplasmacytic infiltration. HCCs with lymphoplasmacytic infiltration (HCC-LIs) show significantly better prognosis in overall (A) and recurrence-free (B) survivals than HCCs with lymphoplasmacytic infiltration (HCC-NLIs).

The median follow-up period for the 544 patients was 52.7 months (range 0.8–168.5 months). Their cumulative overall and recurrence-free survival rates at five years were 42.7% and 19.0%, respectively. Kaplan-Meier analyses showed significantly longer overall and recurrence-free survival of patients in the HCC-LI group than in the HCC-NLI group (*P* < 0.001 each by log-rank tests) (Fig 2). Multivariate Cox regression analyses showed that intratumoral lymphoplasmacytic infiltration, microvascular invasion, intrahepatic metastasis, tumor steatosis, and advanced fibrosis of background liver were independently prognostic of overall survival, whereas intratumoral lymphoplasmacytic infiltration, tumor size, microvascular invasion, intrahepatic metastasis, and advanced fibrosis of background liver were independently prognostic of recurrence-free survival (Table 4).

**Table 4:** Factors prognostic of overall and recurrence-free survival in patients with solitary hepatocellular carcinoma.

	Overall survival						Recurrence-free survival					
	Univariate			Multivariate			Univariate			Multivariate		
	HR	95% CI	*P*	HR	95% CI	*P*	HR	95% CI	*P*	HR	95% CI	*P*
Tumor histology												
Lymphoplasmacytic infiltration absent (vs present)	1.850	1.377–2.518	**< 0.001**	1.731	1.240–2.444	**< 0.001**	1.565	1.271–1.936	**< 0.001**	1.567	1.242–1.983	**< 0.001**
Size ≥ 50mm (vs < 50mm)	1.992	1.509–2.626	**< 0.001**	1.269	0.891–1.810	0.187	1.836	1.495–2.246	**< 0.001**	1.332	1.036–1.714	**0.026**
Histologic grade por (vs well and mod)	2.163	1.580–2.922	**< 0.001**	1.299	0.906–1.845	0.153	1.476	1.147–1.878	**0.003**	0.913	0.684–1.209	0.529
Microvascular invasion present (vs absent)	2.200	1.659–2.936	**< 0.001**	1.642	1.167–2.309	**0.005**	1.767	1.443–2.169	**< 0.001**	1.436	1.128–1.826	**0.003**
Bile duct invasion present (vs absent)	2.113	1.217–3.410	**0.010**	1.442	0.795–2.463	0.218	1.649	1.051–2.455	**0.031**	1.290	0.798–1.997	0.287
Intrahepatic metastasis present (vs absent)	2.574	1.891–3.463	**< 0.001**	1.728	1.217–2.438	**0.002**	2.905	2.294–3.650	**< 0.001**	2.196	1.685–2.844	**< 0.001**
Interstitial fibrosis absent (vs present)	1.306	0.991–1.726	0.057	1.037	0.759–1.425	0.819	1.136	0.928–1.390	0.216	0.963	0.768–1.210	0.746
Neutrophil infiltration absent (vs present)	1.017	0.631–1.765	0.948	1.087	0.634–1.983	0.772	1.162	0806–1.746	0.436	1.209	0.808–1.878	0.367
Necrosis present (vs absent)	1.845	1.391–2.464	**< 0.001**	1.141	0.815–1.601	0.444	1.690	1.377–2.079	**< 0.001**	1.172	0.915–1.500	0.208
Steatosis absent (vs present)	2.005	1.369–3.052	**< 0.001**	1.632	1.090–2.529	**0.016**	1.314	1.030–1.697	**0.027**	1.169	0.900–1.536	0.246
Background histology												
Steatosis absent (vs present)	1.360	1.005–1.867	**0.045**	1.127	0.816–1.577	0.473	1.119	0.903–1.396	0.306	1.091	0.869–1.377	0.458
Advanced fibrosis* present (vs absent)	1.620	1.180–2.269	**0.003**	1.933	1.377–2.762	**< 0.001**	1.386	1.114–1.727	**0.003**	1.553	1.232–1.971	**< 0.001**
Liver cirrhosis present (vs absent)	1.279	0.969–1.687	0.082				1.294	1.053–1.586	**0.014**			

HR, hazard ratio; CI, confidence interval; por, poorly differentiated; well, well differentiated; mod, moderately differentiated.

* Corresponding to stages 3 and 4 in the METAVIR system and NASH-CRN scoring systems.

## Discussion

This study showed that lymphoplasmacytic infiltration in HCC, defined by our simple criteria, was significantly associated with several clinical and pathologic features. Most importantly, this infiltration was independently prognostic for overall and recurrence-free survival, with absence of infiltration showing high hazard ratios for poor prognosis.

The mean serum level of PIVKA2 was lower in the HCC-LI group than in the HCC-NIL group. The previously demonstrated correlation of this tumor marker with tumor size,[22] confirmed in the present study (data not shown), indicated that the lower level of PIVKA2 in the HCC-LI group was largely attributable to the significantly smaller sizes of these tumors. We speculated that the notably better liver function in patients in the HCC-LI group was also related to the smaller sizes of these tumors (larger volume of residual liver). Logistic multivariate analysis, however, showed that intratumoral lymphoplasmacytic infiltration was an independent negative predictor of Child-Pugh stage B and high ICGR15, indicating that liver function was directly associated with the extent of inflammatory infiltration. Presumably, patients with good liver function tend to retain anti-tumor immunity, whereas those with impaired liver function are prone to lose that reactivity. This hypothesis is in agreement with the finding that patients with advanced liver disease are frequently in an immunocompromised state.[23]

The lower incidence of preoperative treatment in the HCC-LI group was likely due to the smaller tumor sizes in these patients, because tumor size and estimated residual liver volume have been identified as major determinants of preoperative treatment in our clinical practice. The higher incidence of tumor necrosis in the HCC-NLI group probably resulted from frequent preoperative treatment.

Pathologically, significantly smaller tumor sizes in the HCC-LI group may reflect inflammatory responses that suppressed tumor growth. Patients in both the HCC-LI and HCC-NLI groups, at least those infected with hepatitis virus, had been regularly and similarly screened for tumor development, suggesting that tumors with inflammatory infiltration grew more slowly. Alternatively, tumors may acquire anti-inflammatory (anti-immune) properties when they grow larger. Further basic studies are needed to determine the mechanism by which inflammatory infiltration influences tumor growth.

The cause of the higher frequency of tumor steatosis in the HCC-LI group may be multifactorial. It may be related to the smaller-size tumors in this group, because small, early-stage HCCs frequently manifest fatty changes.[18] The causal relationship between intracellular lipid accumulation and inflammatory reaction, as observed in patients with non-alcoholic fatty liver disease,[24] suggests that tumor steatosis may have induced inflammatory reaction. A recent study demonstrating that inflammatory cells induced hepatocellular steatosis in a mouse model of non-alcoholic steatohepatitis[25] indicates another possibility.

Frequent interstitial fibrosis of HCC-LIs is quite plausible, as hepatic fibrosis generally results from the immune reactive process.[26] Hepatic stellate cells are thought to play crucial roles in fibrogenesis.[27] These cells also play an important role in fibrosis in HCC, interacting with tumor cells and inflammatory cells.[28]

The present study, analyzing a large cohort of patients with HCC, showed that intratumoral lymphoplasmacytic infiltration was an independent prognostic factor for survival in patients with HCC. This study confirmed the prognostic significance of histologic features previously shown to be prognostic in patients with HCC, including tumor size, histologic grade, microvascular invasion of tumors, and advanced fibrosis of background liver. Of particular note, absence of infiltration showed the second highest hazard ratio for overall survival, after advanced fibrosis of background liver, and the second highest hazard ratio for recurrence-free survival, after intrahepatic metastasis. Additional subgroup analyses of treatment-naïve vs. pretreatment patients (S1–S5 Tables and S1 Fig) showed that HCC-LI cases had better preserved liver function than treatment-naïve cases, while of the pretreatment patients, HCC-LI showed a stronger association with neutrophil infiltration or steatosis. Remarkably, in both the treatment-naïve and pretreatment groups, HCC-LI was associated with better prognosis in terms of both recurrence-free and overall survival as determined using multivariate Cox proportional hazards regression models. These findings indicate that intratumoral lymphoplasmacytic infiltration should be assessed in routine diagnostic practice.

The favorable prognosis of patients with HCC-LI presumably had at least two causes. First, robust antitumor immunity should have inhibited the growth of residual tumor that was not apparent at the time of surgical resection. This mechanism seems responsible for the favorable prognosis of tumors with inflammatory infiltration, which have been shown in cancers of various organs.[9, 29] Additionally, specific conditions associated with hepatocarcinogenesis should be entertained; most patients in this study had advanced chronic liver diseases, and thus were cancer prone. Considering that the rates of liver cirrhosis and advanced fibrosis were similar in the HCC-LI and HCC-NLI groups, patients with HCC-LI, with supposedly healthy immune function, probably exhibited effective antitumorigenic immune reactions to newly developing lesions.

Much remains to be elucidated about the basic mechanisms of tumor immunity. Even the roles of inflammatory cell types have not been sufficiently clarified. In this study, we found that plasma cell infiltration was not infrequently prominent in HCC-LIs, and we therefore described the infiltration as “lymphoplasmacytic infiltration.” Compared with T cells, the roles of B cells in tumor immunity are largely unknown. Although numerous studies have shown that B cells promote rather than inhibit tumor development, recent studies have revealed these cells’ antitumorigenic roles.[30, 31]

Molecular signatures differentiating HCCs with and without infiltration are also of interest. Genetic aspects of tumors may determine the extent of anti-tumor inflammatory reactions. For example, microsatellite instability is strongly associated with lymphocytic infiltration in colorectal cancers.[32] Interestingly, Tan et al.[33] demonstrated that HCCs with marked immune cell infiltrate were associated with the S1 molecular subclass, defined by a transcriptome meta-analysis of global HCC populations. Further studies are needed to determine the detailed relationships between the genetic characteristics of HCC and immune cell reactions.

Determining the optimal definition of lymphoplasmacytic infiltration was one of the most challenging issues in this study. We reviewed several definitions, and adopted a definition that was the most straightforward and reproducible. Two pathologists independently evaluated 82 recent three-year cases, and in 69 (84.1%) of them they were in agreement regarding the presence of lymphocytic infiltration. We believe that this classification scheme is currently reasonable and useful, but further research should evaluate more objective definitions that can be used in daily diagnosis.

## Conclusions

Intratumoral lymphoplasmacytic infiltration was associated with favorable liver function, as shown by lower incidence of Child-Pugh stage B and lower ICGR15 levels, and with several pathologic features, including smaller tumor size, more frequent interstitial fibrosis, and steatosis of the tumor. Most importantly, infiltration was an independent prognostic factor for overall and recurrence-free survival, with absence of infiltration showing high Cox-hazard ratios for poor prognosis.

## Supporting Information

S1 Digital Data Digital slides, including 4 HCCs with lymphoplasmacytic infiltration (HCC-Lis, Case 1–4) and 2 HCCs without lymphoplasmacytic infiltration (HCC-NLIs, Case 5 and 6), are available on our website (http://plaza.umin.ac.jp/~pathdatabase/index.html). Cases 1 and 2 correspond to the cases in Fig 1A and 1B, respectively. Cases 3 and 4 are additional HCC-LI cases with multifocal and diffuse patterns, respectively.
(PDF)

S1 Fig Kaplan-Meier analysis shows significantly better prognosis in overall and recurrence-free survival in both treatment-naïve and pretreatment groups in HCCs with lymphoplasmacytic infiltration (HCC-LIs) than HCCs without lymphoplasmacytic infiltration (HCC-NLIs). (TIF)

S1 Table Demographic and clinical features of treatment-naïve and pretreatment HCC with and without lymphoplasmacytic infiltration. (DOCX)

S2 Table Risk factors for liver dysfunction in treatment-naïve HCC (logistic multivariate analysis). (DOCX)

S3 Table Pathologic features of treatment-naïve and pretreatment HCC with and without lymphoplasmacytic infiltration. (DOCX)

S4 Table Factors prognostic of overall and recurrence-free survival in patients with treatment-naïve solitary HCC. (DOCX)

S5 Table Factors prognostic of overall and recurrence-free survival in patients with pretreatment solitary HCC. (DOCX)
