# Supplementary material for: Histologic Assessment of Intratumoral Lymphoplasmacytic Infiltration Is Useful in Predicting Prognosis of Patients with Hepatocellular Carcinoma
Source: PLoS One. 2016 May 19;11(5):e0155744. doi: 10.1371/journal.pone.0155744 (PMC4873037; doi:10.1371/journal.pone.0155744)
Supplement: S1 Digital Data — Cases 1 and 2 correspond to the cases in Fig 1A and 1B, respectively. Cases 3 and 4 are additional HCC-LI cases with multifocal and diffuse patterns, respectively. (PDF) [file pone.0155744.s001.pdf]

S1. Digital Data.

Digital slides, including 4 HCCs with lymphoplasmacytic infiltration (HCC-Lis, Case 1-4) and 2 HCCs without lymphoplasmacytic infiltration (HCC-NLIs, Case 5 and 6), are available on our website (http://plaza.umin.ac.jp/~pathdatabase/index.html).

Case 1: HCC-LI, multifocal type (used in Fig.1 A). (440MB)

Case 2: HCC-LI, diffuse type (used in Fig.1B). (1208MB)

Case 3: HCC-LI, multifocal type. (913MB)

Case 4: HCC-LI, diffuse type. (949MB)

Case 5: HCC-NLI. (1465MB)

Case 6: HCC-NLI. (1111MB)

Slides were digitalized with a digital slide scanner (Nano Zoomer, Hamamatsu Photonics, Shizuoka, Japan) and uploaded as “ndpi” files. A viewing software is necessary and a free viewer can be downloaded at the following site.

http://www.hamamatsu.com/jp/en/U12388-01.html
