# Supplementary figures and images for: Histologic Assessment of Intratumoral Lymphoplasmacytic Infiltration Is Useful in Predicting Prognosis of Patients with Hepatocellular Carcinoma
Source: PLoS One. 2016 May 19;11(5):e0155744. doi: 10.1371/journal.pone.0155744 (PMC4873037; doi:10.1371/journal.pone.0155744)

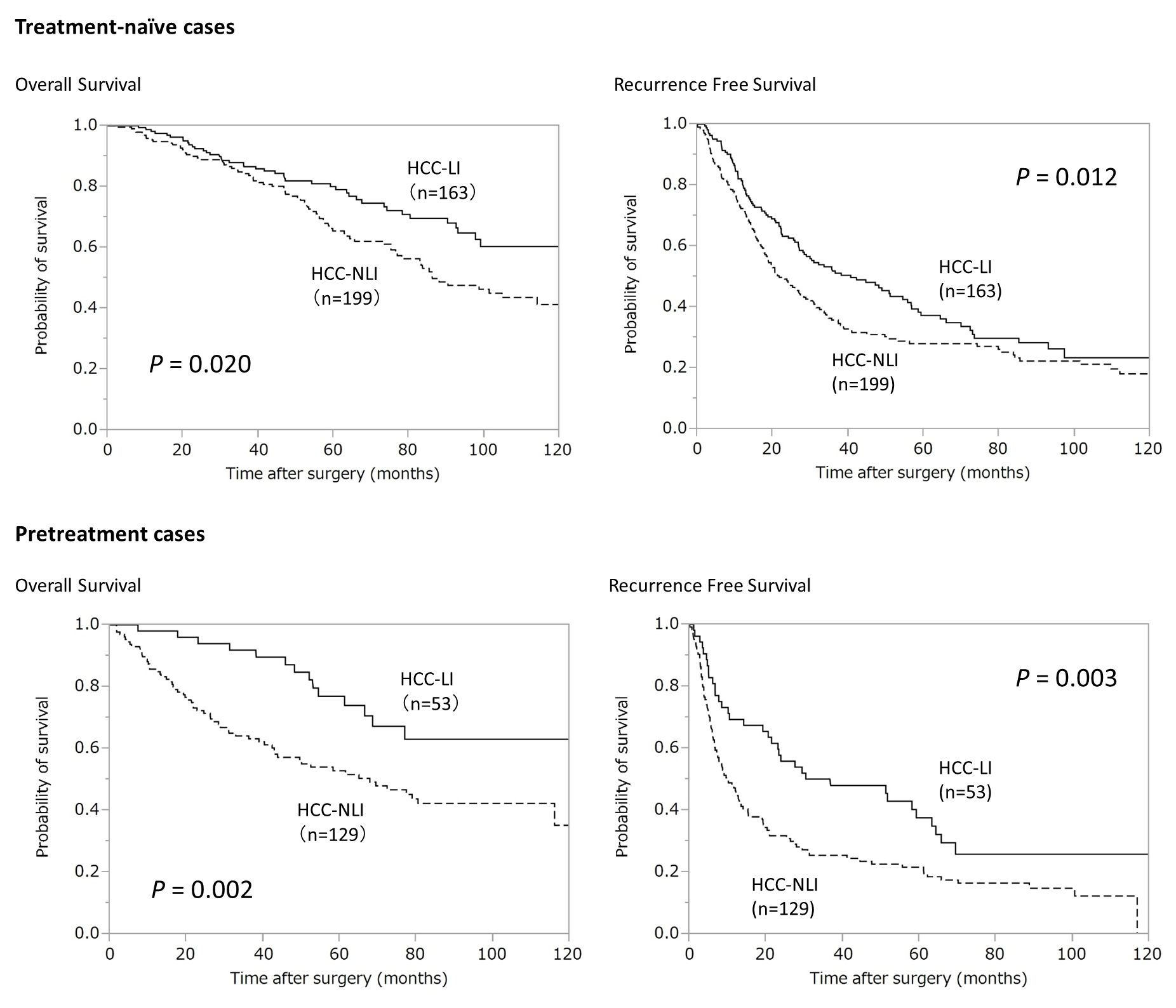

Supplement: S1 Fig — (TIF) [file pone.0155744.s002.TIF]
