# Supplementary material for: Histologic Assessment of Intratumoral Lymphoplasmacytic Infiltration Is Useful in Predicting Prognosis of Patients with Hepatocellular Carcinoma
Source: PLoS One. 2016 May 19;11(5):e0155744. doi: 10.1371/journal.pone.0155744 (PMC4873037; doi:10.1371/journal.pone.0155744)
Supplement: S1 Table — (DOCX) [file pone.0155744.s003.docx]

S1 Table. Demographic and clinical features of treatment-naïve and pretreatment HCC with and without lymphoplasmacytic infiltration.

| Clinical feature | Treatment-naïve | | | |  | Pretreatment | | | |
| --- | --- | --- | --- | --- | --- | --- | --- | --- | --- |
|  | Total N | HCC-LI | HCC-NLI | *P* |  | Total N | HCC-LI | HCC-NLI | *P* |
| Age and Sex |  |  |  |  |  |  |  |  |  |
| Age (years) | 362 |  |  | 0.670 |  | 182 |  |  | 0.325 |
| < 65 | 157 | 73 | 84 |  |  | 80 | 20 | 60 |  |
| > = 65 | 205 | 90 | 115 |  |  | 102 | 33 | 69 |  |
| Sex | 362 |  |  | 0.809 |  | 182 |  |  | 0.836 |
| Male | 271 | 121 | 150 |  |  | 147 | 42 | 105 |  |
| Female | 91 | 42 | 49 |  |  | 35 | 11 | 24 |  |
| Virus Infection |  |  |  |  |  |  |  |  |  |
| HBs-Ag | 362 |  |  | 0.154 |  | 182 |  |  | 0.134 |
| Positive | 76 | 40 | 36 |  |  | 45 | 9 | 36 |  |
| Negative | 286 | 123 | 163 |  |  | 137 | 44 | 93 |  |
| HCV-Ab | 362 |  |  | 0.523 |  | 182 |  |  | 0.871 |
| Positive | 207 | 90 | 117 |  |  | 81 | 23 | 58 |  |
| Negative | 155 | 73 | 82 |  |  | 101 | 30 | 71 |  |
| Diabetes mellitus | 361 |  |  | 0.811 |  | 181 |  |  | 0.249 |
| Present | 96 | 42 | 54 |  |  | 43 | 16 | 27 |  |
| Absent | 265 | 121 | 144 |  |  | 138 | 37 | 101 |  |
| History of heavy alcohol consumption  (≥ 80g per day) | 356 |  |  | 0.889 |  | 181 |  |  | 0.302 |
| Present | 62 | 29 | 33 |  |  | 35 | 13 | 22 |  |
| Absent | 294 | 134 | 160 |  |  | 146 | 40 | 106 |  |
| Serum tumor marker |  |  |  |  |  |  |  |  |  |
| AFP [ng/ml, median (IQR)] | 362 | 21 (323) | 14(96) | 0.530 |  | 182 | 62 (1141) | 42(855) | 0.150 |
| PIVKA-2 [mAU/ml, median (IQR)] | 357 | 63 (404) | 63 (720) | 0.234 |  | 182 | 345 (3941) | 599 (8087) | 0.148 |
| CEA [ng/ml, median (IQR)] | 324 | 3.0 (2.9) | 3.8 (3.6) | **0.011** |  | 173 | 3.7 (3.1) | 3.0 (2.3) | 0.962 |
| CA19-9 [U/ml, median (IQR)] | 319 | 17 (23) | 18 (23) | 0.970 |  | 171 | 16 (13) | 14 (17) | 0.067 |
| Liver Function |  |  |  |  |  |  |  |  |  |
| Child-Pugh stage | 362 |  |  | **0.009** |  | 182 |  |  | 0.181 |
| A | 325 | 154 | 171 |  |  | 154 | 48 | 106 |  |
| B | 37 | 9 | 28 |  |  | 28 | 5 | 23 |  |
| ICGR15 [%, mean ± SD] | 358 | 13.6 ± 7.9 | 16.3 ± 10.5 | **0.008** |  | 181 | 12.4 ± 6.4 | 14.9 ± 8.5 | 0.052 |
| Total protein [U/ml, mean ± SD] | 362 | 7.0 ± 0.5 | 7.0 ± 0.6 | 0.498 |  | 182 | 7.2 ± 0.6 | 7.1 ± 0.6 | 0.392 |
| Albumin [g/dl, mean ± SD] | 362 | 3.8 ± 0.4 | 3.7 ± 0.4 | **0.003** |  | 182 | 3.7 ± 0.4 | 3.5 ± 0.4 | **0.034** |
| AST [IU/l, mean ± SD] | 362 | 43.4 ± 23.5 | 53.0 ± 41.2 | **0.008** |  | 182 | 40.3 ± 23.7 | 48.8 ± 27.9 | 0.068 |
| ALT [IU/l, mean ± SD] | 362 | 45.3 ± 29.3 | 48.8 ± 30.6 | 0.279 |  | 182 | 36.0 ± 26.9 | 46.6 ± 42.2 | 0.112 |
| Total bilirubin [mg/dl, mean ± SD] | 362 | 0.78 ± 0.34 | 0.80 ± 0.28 | 0.367 |  | 182 | 0.61 ± 0.21 | 0.71 ± 0.29 | **0.028** |

HCC-LI, hepatocellular carcinoma with lymphoplasmacytic infiltration; HCC-NLI, hepatocellular carcinoma with no lymphoplasmacytic infiltration; AFP, alpha fetoprotein; PIVKA-2, protein induced by vitamin K absence or antagonist 2; CEA, carcinoembryonic antigen; CA19-9, carbohydrate antigen 19-9; ICGR15, indocyanine green retention rate at 15 minutes; AST, aspartate aminotransferase; ALT, alanine aminotransferase; IQR, interquartile range; SD, standard deviation
