# Supplementary material for: Histologic Assessment of Intratumoral Lymphoplasmacytic Infiltration Is Useful in Predicting Prognosis of Patients with Hepatocellular Carcinoma
Source: PLoS One. 2016 May 19;11(5):e0155744. doi: 10.1371/journal.pone.0155744 (PMC4873037; doi:10.1371/journal.pone.0155744)
Supplement: S2 Table — (DOCX) [file pone.0155744.s004.docx]

S2 Table. Risk factors for liver dysfunction in treatment-naïve HCC (logistic multivariate analysis) .

|  | Child Pugh stage B | | | | |  | ICG15 >15% | | | | |
| --- | --- | --- | --- | --- | --- | --- | --- | --- | --- | --- | --- |
|  | HR | 95% CI | | | *P*-value |  | HR | 95% CI | | | *P*-value |
| Tumor histology |  |  |  |  |  |  |  |  |  |  |  |
| Lymphoplasmacytic infiltration absent (vs present) | 2.866 | 1.234 | - | 7.221 | **0.014** |  | 1.601 | 0.962 | - | 2.683 | 0.070 |
| Tumor size < 50mm (vs ≥ 50mm) | 1.647 | 0.623 | - | 4.948 | 0.325 |  | 1.073 | 0.572 | - | 2.030 | 0.826 |
| Histologic grade well and mod (vs por) | 1.959 | 0.681 | - | 6.602 | 0.221 |  | 1.118 | 0.552 | - | 2.297 | 0.758 |
| Microvascular invasion absent (vs present) | 1.061 | 0.564 | - | 2.019 | 0.854 |  | 0.949 | 0.559 | - | 1.610 | 0.845 |
| Bile duct invasion absent (vs present) | 0.993 | 0.147 | - | 20.000 | 0.914 |  | 0.959 | 0.231 | - | 4.340 | 0.955 |
| Intrahepatic metastasis absent (vs present) | 2.063 | 0.612 | - | 9.580 | 0.259 |  | 0.966 | 0.465 | - | 2.043 | 0.966 |
| Interstitial fibrosis absent (vs present) | 0.767 | 0.345 | - | 1.708 | 0.515 |  | 1.416 | 0.842 | - | 2.389 | 0.190 |
| Neutrophil infiltration absent (vs present) | 1.338 | 0.331 | - | 9.101 | 0.711 |  | 0.478 | 0.187 | - | 1.221 | 0.122 |
| Necrosis present (vs absent) | 2.479 | 1.106 | - | 5.612 | **0.028** |  | 0.557 | 0.321 | - | 0.955 | **0.033** |
| Steatosis present (vs absent) | 1.110 | 0.447 | - | 2.576 | 0.815 |  | 1.475 | 0.940 | - | 2.319 | 0.257 |
| Background histology |  |  |  |  |  |  |  |  |  |  |  |
| Steatosis absent (vs present) | 1.142 | 0.525 | - | 2.574 | 0.742 |  | 0.730 | 0.423 | - | 1.258 | 0.427 |
| Advanced fibrosis present (vs absent) | 5.540 | 1.877 | - | 23.773 | **< 0.001** |  | 4.391 | 2.511 | - | 8.002 | **< 0.001** |

HR, hazard ratio; CI, confidence interval; por, poorly differentiated; well, well differentiated; mod, moderately differentiated.

Advanced fibrosis corresponds to stages 3 and 4 in the METAVIR system and NASH-CRN scoring systems.
