# Supplementary material for: Histologic Assessment of Intratumoral Lymphoplasmacytic Infiltration Is Useful in Predicting Prognosis of Patients with Hepatocellular Carcinoma
Source: PLoS One. 2016 May 19;11(5):e0155744. doi: 10.1371/journal.pone.0155744 (PMC4873037; doi:10.1371/journal.pone.0155744)
Supplement: S3 Table — (DOCX) [file pone.0155744.s005.docx]

S3 Table. Pathologic features of treatment-naïve and pretreatment HCC with and without lymphoplasmacytic infiltration.

| Pathologic feature | Treatment-naïve | | | |  | Pretreatment | | | |
| --- | --- | --- | --- | --- | --- | --- | --- | --- | --- |
|  | Total N | HCC-LI | HCC-NLI | *P* |  | Total N | HCC-LI | HCC-NLI | *P* |
| **Tumor** |  |  |  |  |  |  |  |  |  |
| Size (mm) |  |  |  | 0.213 |  |  |  |  | **0.0498** |
| < 50 | 277 | 130 | 147 |  |  | 55 | 22 | 33 |  |
| ≥ 50 | 85 | 33 | 52 |  |  | 127 | 31 | 96 |  |
| Histologic grade |  |  |  | 0.723 |  |  |  |  | 0.245 |
| Well differentiated | 65 | 28 | 37 |  |  | 11 | 4 | 7 |  |
| Moderately differentiated | 241 | 112 | 129 |  |  | 122 | 39 | 83 |  |
| Poorly differentiated | 56 | 23 | 33 |  |  | 49 | 10 | 39 |  |
| Microvascular invasion |  |  |  | 0.072 |  |  |  |  | 0.166 |
| Present | 163 | 82 | 81 |  |  | 123 | 40 | 83 |  |
| Absent | 199 | 81 | 118 |  |  | 59 | 13 | 46 |  |
| Bile duct invasion |  |  |  | 0.355 |  |  |  |  | 0.171 |
| Present | 10 | 6 | 4 |  |  | 18 | 8 | 10 |  |
| Absent | 352 | 157 | 195 |  |  | 164 | 45 | 119 |  |
| Intrahepatic metastasis |  |  |  | 0.534 |  |  |  |  | 0.736 |
| Present | 48 | 24 | 24 |  |  | 65 | 20 | 45 |  |
| Absent | 314 | 139 | 175 |  |  | 117 | 33 | 84 |  |
| Neutrophil infiltration |  |  |  | 0.146 |  |  |  |  | **< 0.001** |
| Present | 25 | 15 | 10 |  |  | 14 | 12 | 2 |  |
| Absent | 337 | 148 | 189 |  |  | 168 | 41 | 127 |  |
| Necrosis |  |  |  | 0.335 |  |  |  |  | 0.498 |
| Present | 151 | 63 | 88 |  |  | 154 | 43 | 111 |  |
| Absent | 211 | 100 | 111 |  |  | 28 | 10 | 18 |  |
| Interstitial fibrosis |  |  |  | **< 0.001** |  |  |  |  | **< 0.001** |
| Present | 191 | 122 | 69 |  |  | 84 | 43 | 41 |  |
| Absent | 171 | 41 | 130 |  |  | 98 | 10 | 88 |  |
| Steatosis |  |  |  | 0.116 |  |  |  |  | **< 0.001** |
| Present | 92 | 48 | 44 |  |  | 32 | 18 | 14 |  |
| Absent | 270 | 115 | 155 |  |  | 150 | 35 | 115 |  |
| **Background liver** |  |  |  |  |  |  |  |  |  |
| Steatosis (>5%) |  |  |  | 1.000 |  |  |  |  | 0.571 |
| Present | 128 | 58 | 70 |  |  | 45 | 15 | 30 |  |
| Absent | 234 | 105 | 129 |  |  | 137 | 38 | 99 |  |
| Advanced fibrosis* |  |  |  | 0.168 |  |  |  |  | 0.319 |
| Present | 252 | 107 | 145 |  |  | 108 | 28 | 80 |  |
| Absent | 110 | 56 | 54 |  |  | 74 | 25 | 49 |  |

HCC-LI, hepatocellular carcinoma with lymphoplasmacytic infiltration; HCC-NLI, hepatocellular carcinoma with no lymphoplasmacytic infiltration.

* Corresponding to stages 3 and 4 in the METAVIR system and NASH-CRN scoring systems
