# Supplementary material for: Histologic Assessment of Intratumoral Lymphoplasmacytic Infiltration Is Useful in Predicting Prognosis of Patients with Hepatocellular Carcinoma
Source: PLoS One. 2016 May 19;11(5):e0155744. doi: 10.1371/journal.pone.0155744 (PMC4873037; doi:10.1371/journal.pone.0155744)
Supplement: S4 Table — (DOCX) [file pone.0155744.s006.docx]

S4 Table. Factors prognostic of overall and recurrence-free survival in patients with treatment-naïve solitary HCC

|  | Overall survival | | | | | | |  | Recurrence-free survival | | | | | | |
| --- | --- | --- | --- | --- | --- | --- | --- | --- | --- | --- | --- | --- | --- | --- | --- |
|  | Univariate | | |  | Multivariate | | |  | Univariate | | |  | Multivariate | | |
|  | HR | 95% CI | *P* |  | HR | 95% CI | *P* |  | HR | 95% CI | *P* |  | HR | 95% CI | *P* |
| Tumor histology |  |  |  |  |  |  |  |  |  |  |  |  |  |  |  |
| Lymphoplasmacytic infiltration absent (vs present) | 1.535 | 1.072–2.224 | **0.019** |  | 1.500 | 1.024–2.216 | **0.037** |  | 1.386 | 1.075–1.793 | **0.012** |  | 1.379 | 1.065–1.792 | **0.015** |
| Size ≥ 50mm (vs < 50mm) | 1.899 | 1.277–2.770 | **0.002** |  | 1.286 | 0.833–1.956 | 0.251 |  | 1.642 | 1.225–2.172 | **0.001** |  | 1.391 | 1.005–1.905 | **0.046** |
| Histologic grade por  (vs well and mod) | 2.101 | 1.350–3.168 | **0.001** |  | 1.567 | 0.965–2.481 | 0.069 |  | 1.472 | 1.049–2.021 | **0.026** |  | 1.103 | 0.758–1.578 | 0.601 |
| Microvascular invasion present  (vs absent) | 1.933 | 1.353–2.763 | **< 0.001** |  | 1.496 | 0.989–2.251 | 0.056 |  | 1.623 | 1.261–2.088 | **< 0.001** |  | 1.302 | 0.981–1.722 | 0.067 |
| Bile duct invasion present  (vs absent) | 4.350 | 1.684–9.216 | **0.004** |  | 4.118 | 1.547–9.178 | **0.007** |  | 2.011 | 0.909–3.808 | 0.081 |  |  |  |  |
| Intrahepatic metastasis present  (vs absent) | 2.102 | 1.278–3.295 | **0.004** |  | 1.542 | 0.903–2.534 | 0.110 |  | 3.376 | 2.395–4.655 | **< 0.001** |  | 2.891 | 2.011–4.075 | **< 0.001** |
| Interstitial fibrosis absent  (vs present) | 1.432 | 1.007–2.046 | **0.046** |  | 1.394 | 0.952-2.059 | 0.088 |  | 1.184 | 0.921–1.523 | 0.188 |  |  |  |  |
| Neutrophil infiltration absent  (vs present) | 0.799 | 0.450–1.576 | 0.492 |  |  |  |  |  | 0.954 | 0.609–1.563 | 0.817 |  |  |  |  |
| Necrosis present (vs absent) | 1.783 | 1.252–2.535 | **0.001** |  | 1.340 | 0.907–1.977 | 0.141 |  | 1.480 | 1.148–1.904 | **0.003** |  | 1.146 | 0.854–1.532 | 0.361 |
| Steatosis absent (vs present) | 1.511 | 0.979–2.434 | 0.063 |  |  |  |  |  | 1.242 | 0.928–1.689 | 0.147 |  |  |  |  |
| Background histology |  |  |  |  |  |  |  |  |  |  |  |  |  |  |  |
| Steatosis absent (vs present) | 1.310 | 0.900–1.946 | 0.160 |  |  |  |  |  | 0.983 | 0.758–1.284 | 0.900 |  |  |  |  |
| Advanced fibrosis* present  (vs absent) | 1.354 | 0.897–2.115 | 0.153 |  |  |  |  |  | 1.599 | 1.197–2.171 | **0.001** |  | 1.706 | 1.266–2.335 | **< 0.001** |
| Liver cirrhosis present (vs absent) | 1.295 | 0.911–1.844 | 0.149 |  |  |  |  |  | 1.437 | 1.116–1.849 | **0.001** |  |  |  |  |

HR, hazard ratio; CI, confidence interval; por, poorly differentiated; well, well differentiated; mod, moderately differentiated.

* Corresponding to stages 3 and 4 in the METAVIR system and NASH-CRN scoring systems.
