# Supplementary material for: Histologic Assessment of Intratumoral Lymphoplasmacytic Infiltration Is Useful in Predicting Prognosis of Patients with Hepatocellular Carcinoma
Source: PLoS One. 2016 May 19;11(5):e0155744. doi: 10.1371/journal.pone.0155744 (PMC4873037; doi:10.1371/journal.pone.0155744)
Supplement: S5 Table — (DOCX) [file pone.0155744.s007.docx]

S5 Table. Factors prognostic of overall and recurrence-free survival in patients with pretreatment solitary HCC

|  | Overall survival | | | | | | |  | Recurrence-free survival | | | | | | |
| --- | --- | --- | --- | --- | --- | --- | --- | --- | --- | --- | --- | --- | --- | --- | --- |
|  | Univariate | | |  | Multivariate | | |  | Univariate | | |  | Multivariate | | |
|  | HR | 95% CI | *P* |  | HR | 95% CI | *P* |  | HR | 95% CI | *P* |  | HR | 95% CI | *P* |
| Tumor histology |  |  |  |  |  |  |  |  |  |  |  |  |  |  |  |
| Lymphoplasmacytic infiltration absent (vs present) | 2.469 | 1.428–4.587 | **< 0.001** |  | 2.499 | 1.374–4.875 | **0.002** |  | 1.798 | 1.234–2.683 | **0.002** |  | 2.177 | 1.467–3.302 | **< 0.001** |
| Size ≥ 50mm (vs < 50mm) | 1.863 | 1.137–3.185 | **0.013** |  | 1.001 | 0.555–1.855 | 0.996 |  | 1.801 | 1.248–2.653 | **0.002** |  | 1.100 | 0.716–1.725 | 0.668 |
| Histologic grade por  (vs well and mod) | 2.030 | 1.267–3.191 | **0.004** |  | 1.187 | 0.704–1.975 | 0.516 |  | 1.304 | 0.884–1.885 | 0.176 |  | 1.120 | 0.749–1.700 | 0.588 |
| Microvascular invasion present  (vs absent) | 2.493 | 1.498–4.362 | **0.003** |  | 2.322 | 1.220–4.554 | **0.010** |  | 1.805 | 1.258–2.639 | **0.001** |  | 1.646 | 1.067–2.562 | **0.024** |
| Bile duct invasion present  (vs absent) | 1.302 | 0.628–2.418 | 0.452 |  |  |  |  |  | 1.279 | 0.717-2.118 | 0.385 |  |  |  |  |
| Intrahepatic metastasis present  (vs absent) | 2.609 | 1.671–4.079 | **< 0.001** |  | 1.942 | 1.166–3.278 | **0.011** |  | 2.235 | 1.572–3.157 | **< 0.001** |  | 1.859 | 1.260–2.742 | **0.002** |
| Interstitial fibrosis absent  (vs present) | 1.075 | 0.691-1.687 | 0.748 |  |  |  |  |  | 0.978 | 0.699–1.373 | 0.897 |  |  |  |  |
| Neutrophil infiltration absent  (vs present) | 1.560 | 0.695–4.460 | 0.307 |  |  |  |  |  | 1.718 | 0.921–3.659 | 0.093 |  |  |  |  |
| Necrosis present (vs absent) | 1.594 | 0.876–3.199 | 0.132 |  |  |  |  |  | 1.924 | 1.200–3.274 | **0.006** |  | 1.444 | 0.845–2.578 | 0.180 |
| Steatosis absent (vs present) | 3.771 | 1.683–10.763 | **< 0.001** |  | 1.980 | 0.818-5.934 | 0.138 |  | 1.400 | 0.906–2.268 | 0.134 |  |  |  |  |
| Background histology |  |  |  |  |  |  |  |  |  |  |  |  |  |  |  |
| Steatosis absent (vs present) | 1.319 | 0.796–2.298 | 0.292 |  |  |  |  |  | 1.354 | 0.923-2.043 | 0.124 |  |  |  |  |
| Advanced fibrosis* present  (vs absent) | 2.287 | 1.402–3.891 | **< 0.001** |  | 2.592 | 1.549-4.510 | **< 0.001** |  | 1.260 | 0.896–1.789 | 0.185 |  |  |  |  |
| Liver cirrhosis present (vs absent) | 1.438 | 0.903–2.258 | 0.124 |  |  |  |  |  | 1.211 | 0.839–1.726 | 0.301 |  |  |  |  |

HR, hazard ratio; CI, confidence interval; por, poorly differentiated; well, well differentiated; mod, moderately differentiated.

* Corresponding to stages 3 and 4 in the METAVIR system and NASH-CRN scoring systems.
